# Postoperative Blood Transfusion as an Independent Predictor of Morbidity and Mortality After Carotid Endarterectomy: A Single-Centre Retrospective Cohort Study

**DOI:** 10.3390/jcm15135269

**Published:** 2026-07-06

**Authors:** Maria Antonia Ibáñez, Daniel Gómez-Alonso, Carlos Vaquero, Enrique María San Norberto

**Affiliations:** 1 Angiology and Vascular Surgery, Valladolid University Hospital, 47003 Valladolid, Spain; 2 General and Digestive System Surgery, Valladolid University Hospital, 47003 Valladolid, Spain

**Keywords:** carotid endarterectomy, blood transfusion, postoperative mortality, anaemia, peripheral arterial disease, patient blood management

## Abstract

**Objective**: To determine the prevalence and independent predictors of postoperative blood transfusion following carotid endarterectomy (CEA) and to quantify its association with in-hospital morbidity and 30-day mortality. **Methods**: A retrospective, single-centre cohort study was conducted. All consecutive patients undergoing CEA for carotid artery stenosis for two years were included without exclusion criteria. Preoperative and serial postoperative haemoglobin (Hb) and platelet values were collected at 6, 12, 24, and 48 h after surgery, together with demographic variables, cardiovascular risk factors, neurological presentation, anaesthetic risk (ASA classification), operative details, transfusion characteristics, postoperative complications, length of stay, and 30-day mortality. Red cell concentrate was indicated when postoperative Hb was ≤9 g/dL and platelet concentrate when postoperative platelets fell below 100 × 10^3^/mm^3^. The primary outcome was 30-day mortality. Univariate and multivariate linear regression analyses were used to identify independent predictors of mortality and transfusion need. Statistical significance was set at *p* < 0.05; analyses were performed with SPSS v30.0. **Results**: A total of 182 patients underwent CEA; 87.4% were male with a mean age of 71.84 years. The 30-day mortality rate was 3.8% (*n* = 7). Blood transfusion was required in 8.2% of patients (*n* = 15). On multivariate analysis, postoperative transfusion (*p* = 0.001) and postoperative complications (*p* = 0.001) were the only independent predictors of mortality, with transfusion conferring an approximately five-fold increase in mortality risk (relative risk [RR] 4.99; 95% confidence interval [CI] 0.88–28.24). Transfused patients had significantly higher complication rates (60.0% vs. 13.8%; *p* < 0.001) and longer total hospital stays (11.2 ± 14.9 vs. 4.9 ± 2.3 days; *p* = 0.001). On multivariate analysis, independent predictors of transfusion were low preoperative Hb (*p* < 0.001), peripheral arterial disease (PAD; RR 5.09, 95% CI 1.70–15.21; *p* = 0.002), postoperative complications (*p* = 0.004), and prolonged hospital stay (*p* < 0.001). **Conclusions**: Postoperative blood transfusion is an independent risk factor for mortality after CEA, multiplying mortality risk approximately five-fold. Low preoperative Hb and peripheral arterial disease are the principal preoperative predictors of transfusion requirement. These findings underscore the importance of systematic preoperative anaemia optimisation and the application of evidence-based restrictive transfusion thresholds in patients undergoing carotid surgery.

## 1. Introduction

Carotid endarterectomy remains the gold-standard surgical treatment for significant internal carotid artery stenosis and is among the most frequently performed vascular procedures worldwide. Its efficacy in reducing ipsilateral ischaemic stroke has been established in the landmark NASCET, ECST, and ACAS randomised controlled trials, provided perioperative stroke and mortality rates are maintained below 3% for asymptomatic and 6% for symptomatic lesions [1,2,3]. The progressive adoption of standardised patient pathways, patch angioplasty, and improved anaesthetic monitoring has steadily improved outcomes; however, postoperative haematological decline remains an inherent consequence of CEA, and a clinically relevant subset of patients require allogeneic blood transfusion. Published transfusion rates after CEA vary widely—from less than 1% in selected tertiary-centre series to over 8% in real-world cohorts with broader indications and higher comorbidity burden—reflecting the absence of standardised transfusion thresholds across institutions [4,5].

Transfusion carries well-documented risks including acute haemolytic reactions, alloimmunisation, transfusion-related acute lung injury, febrile non-haemolytic reactions, and immunomodulation [6]. Beyond these immunological complications, stored allogeneic red cells undergo progressive biochemical deterioration during storage—the so-called storage lesion—characterised by depletion of 2,3-diphosphoglycerate and adenosine triphosphate, oxidative membrane damage, and the accumulation of bioactive lipids and proinflammatory cytokines [7]. These changes impair erythrocyte deformability and oxygen delivery at the tissue level, and upon transfusion may trigger systemic endothelial inflammation and microcirculatory dysfunction. Evidence from cardiac and major vascular surgery consistently identifies allogeneic blood transfusion as an independent predictor of adverse postoperative outcomes, including increased mortality, myocardial infarction, cerebrovascular events, respiratory failure, and prolonged intensive care stay [8,9]. In the specific context of CEA, Rubinstein et al. demonstrated that intraoperative transfusion of even one or two units of packed red cells was associated with a five-fold increase in perioperative stroke risk, suggesting that the cerebrovascular consequences of transfusion after carotid surgery may be disproportionately severe compared with other vascular procedures [5].

Patients undergoing CEA represent a distinctly vulnerable group in whom both perioperative anaemia and its correction may be hazardous. Their high burden of generalised atherosclerosis, frequent coexistent ischaemic heart disease and chronic kidney disease, and the haemodynamic sensitivity of cerebral perfusion to changes in blood viscosity and oxygen-carrying capacity create a narrow therapeutic window. The optimal haemoglobin threshold at which to initiate transfusion in this population remains debated, and practice in many centres continues to be guided by clinician discretion rather than a validated, disease-specific protocol. The debate between liberal and restrictive transfusion strategies in high-cardiovascular-risk patients remains unresolved, with major randomised trials reporting conflicting findings on the optimal trigger for intervention [10,11,12]. Preoperative anaemia—itself an independent predictor of postoperative morbidity and mortality across surgical specialties—is highly prevalent in this group owing to the systemic burden of atherosclerotic disease and its associated comorbidities, yet remains undertreated in the pre-admission setting [13,14]. Systematic identification of transfusion predictors and rigorous quantification of its prognostic impact are therefore essential prerequisites for implementing structured patient blood management programmes in CEA [15].

The aims of this study were to determine the prevalence and preoperative predictors of postoperative blood transfusion after CEA, considering postoperative transfusion as a potential independent risk factor for the development of postoperative complications.

## 2. Methods

### 2.1. Study Design and Setting

A retrospective cohort study was conducted at the Service of Angiology and Vascular Surgery, Valladolid University Hospital, Spain. All consecutive patients who underwent CEA for carotid artery stenosis for two years were included. No exclusion criteria were applied, given that no follow-up beyond the index admission was required for the study endpoints. Patient data were anonymised prior to analysis. This study was conducted in accordance with the Declaration of Helsinki; patient confidentiality was maintained throughout by substituting clinical record numbers with anonymised assignment codes, and was approved by each institutional review board (PI-26-2011, Valladolid Ethics Committee for Clinical Investigation).

### 2.2. Data Collection

Data were retrieved from clinical records and the hospital’s electronic clinical report management system. Demographic information—age and sex—was recorded for all patients, together with a comprehensive assessment of cardiovascular risk factors including hypertension, dyslipidaemia, diabetes mellitus, and smoking status (active or ex-smoker). Relevant comorbidities collected comprised ischaemic heart disease, peripheral arterial disease (PAD), chronic obstructive pulmonary disease, chronic kidney disease (in accordance with KDIGO guidelines), hepatic insufficiency, and a history of prior contralateral CEA. Neurological presentation was classified as asymptomatic, amaurosis fugax, transient ischaemic attack (TIA), or established stroke, with documentation of whether neurological recovery was complete at the time of surgery. The degree of ipsilateral and contralateral carotid stenosis was recorded alongside the diagnostic modality used to confirm and characterise the lesion, which included CT angiography, digital subtraction angiography, MR angiography, and colour duplex ultrasonography. Preoperative anaesthetic risk was graded using the ASA physical status classification. Operative variables documented were anaesthetic technique (general or locoregional) and the material used for arteriotomy closure. Haematological indices—haemoglobin (Hb, g/dL) and platelet count (×10^3^/mm^3^)—were measured preoperatively and at 6, 12, 24, and 48 h postoperatively. Transfusion data included indication, blood product type, number of units administered, and timing relative to surgery. Patients requiring pre- or intraoperative transfusion were excluded from the analysis (no patient operated on during the study period required them). Duration of stay in the recovery unit and total in-hospital stay in days were recorded. Postoperative complications captured were cervical haematoma not requiring surgical intervention, cervical bleeding requiring operative re-exploration, ischaemic stroke, acute myocardial infarction, peripheral nerve injury, and cerebral reperfusion syndrome. The primary outcome was 30-day or in-hospital mortality. Antiplatelet therapy was indicated following the ESVS guidelines [16], although only 96.7% of patients complied with the prescribed antiplatelet therapy.

### 2.3. Transfusion Protocol

Red cell concentrate was transfused when postoperative Hb was ≤9 g/dL at any scheduled time point. One unit was expected to raise Hb by approximately 0.8 g/dL in a 70 kg patient. Platelet concentrate was indicated when the postoperative platelet count fell below 100 × 10^3^/mm^3^. In practice, the final transfusion decision rested with the attending clinician in the recovery unit, as no formal institutional restrictive or liberal protocol was operative during the study period.

### 2.4. Statistical Analysis

Descriptive statistics are presented as mean ± standard deviation (SD) for continuous variables and as frequency (percentage) for categorical variables. Normality was assessed with the Kolmogorov–Smirnov and Shapiro–Wilk tests. Comparisons between groups were performed using Student’s *t*-test or the Mann–Whitney U test for continuous variables, and the chi-squared test or Fisher’s exact test for categorical variables, as appropriate. Multivariate linear regression identified independent predictors of mortality and blood transfusion. Relative risk (RR) with 95% confidence intervals was calculated for significant transfusion predictors. Statistical significance was set at *p* < 0.05; all analyses were performed using SPSS v30.0.

## 3. Results

### 3.1. Patient Characteristics

A total of 182 patients underwent CEA during the study period. The cohort was predominantly male (87.4%; *n* = 159) with a mean age of 71.84 years (median: 74, range: 48–88). The most prevalent cardiovascular risk factors were hypertension (64.3%), dyslipidaemia (54.4%), and diabetes mellitus (38.5%); 59.9% had a smoking history (active 25.3%; ex-smokers 34.6%). Concomitant ischaemic heart disease and PAD were each documented in 25.8% of patients. Chronic obstructive pulmonary disease was present in 10.4%, chronic kidney disease in 8.2%, and hepatic insufficiency in 3.3%. A previous contralateral CEA had been performed in 13.7% of the cohort. Full baseline characteristics are summarised in Table 1.

From a neurological standpoint, 35.7% were asymptomatic, 33.0% had a prior established stroke, 24.7% presented with TIA, and 6.6% with amaurosis fugax; neurological recovery was complete in 46.2% at the time of surgery. CT angiography was the predominant diagnostic modality (64.3%), followed by digital subtraction angiography (27.5%), MR angiography (7.1%), and colour duplex ultrasonography (1.1%). Ipsilateral carotid stenosis was severe (70–99%) in 88.5% and moderate to significant (50–70%) in 11.5%. Contralateral carotid occlusion was present in 11.0%. Anaesthetic risk was ASA III in 78.6%, ASA II in 18.7%, and ASA IV in 2.7%, reflecting the high systemic comorbidity of the cohort. All procedures were performed under general anaesthesia, with Dacron patch closure in 87.4% of cases.

### 3.2. Haematological Values and Postoperative Course

Mean preoperative Hb was 13.55 ± 2.16 g/dL and the mean platelet count was 221.25 ± 69.78 × 10^3^/mm^3^. Postoperatively, mean Hb declined significantly to 11.83 ± 6.88 g/dL (*p* = 0.032), whilst the mean platelet count fell to 197.02 ± 64.73 × 10^3^/mm^3^, a reduction that did not reach statistical significance (*p* = 0.352). This postoperative haematological decline is consistent with the expected haemodilution and blood loss associated with arterial reconstruction under general anaesthesia. The mean duration of stay in the recovery unit was 1.45 ± 1.74 days and total in-hospital stay was 5.45 ± 4.99 days.

Postoperative complications occurred in 32 patients (17.6%): surgical cervical bleeding requiring operative re-exploration in 12 patients (6.6%), ischaemic stroke in 11 patients (6.0%), acute myocardial infarction in 3 patients (1.6%), cervical haematoma not requiring surgical drainage in 3 patients (1.6%), peripheral nerve injury in 2 patients (1.1%), and cerebral reperfusion syndrome in 1 patient (0.5%). The most common single complication requiring re-intervention was haemorrhage at the operative site, which occurred more frequently in the transfused group, suggesting a bidirectional relationship between bleeding and transfusion need. The proportion of patients experiencing no complication was 82.4%.

### 3.3. Mortality

The 30-day postoperative mortality rate was 3.8% (7 of 182 patients: three due to ischaemic heart disease, two strokes, one pneumonia, and one postoperative pulmonary embolism). Analysis of preoperative variables revealed no statistically significant difference between survivors and non-survivors with respect to sex, diabetes mellitus, hypertension, dyslipidaemia, smoking status, ischaemic heart disease, COPD, chronic kidney disease, hepatic insufficiency, PAD, prior contralateral CEA, neurological presentation, degree of ipsilateral or contralateral stenosis, diagnostic modality, preoperative haemoglobin, preoperative platelet count, ASA class, or closure material. Notably, all seven patients who died had been classified as ASA III, and none were ASA IV, reflecting the paradox that ASA IV patients may have been subject to more cautious selection criteria.

All patients who died experienced at least one postoperative complication (100% vs. 14.3% in survivors; *p* < 0.001), confirming that complications were the proximate clinical antecedent of death in every case. Deceased patients had a significantly longer stay in the recovery unit (3.00 vs. 1.39 days; *p* = 0.001) and a higher transfusion rate (28.6% vs. 7.4%; *p* = 0.046). Postoperative Hb and platelet values did not differ significantly between survivors and non-survivors. On multivariate regression analysis, the two independent predictors of mortality were postoperative transfusion (*p* = 0.001) and the occurrence of postoperative complications (*p* = 0.001). Postoperative transfusion was associated with an approximately five-fold increase in mortality risk in the multivariate model (RR 4.99; 95% CI 0.88–28.24), though the wide confidence interval crossing unity reflects substantial statistical uncertainty arising from the small number of mortality events (*n* = 7) and should be interpreted as exploratory (Table 2).

### 3.4. Blood Transfusion

Blood transfusion was required for 15 patients (8.2%): 12 received red cell concentrates only (6.6%) and three received both red cell and platelet concentrates (1.6%). The most common transfusion volume was two units of red cell concentrate (nine patients; 4.9%). Forty percent of transfusions were administered within the first 24 h postoperatively; the remainder was administered after 24 h. Transfused patients had significantly higher complication rates (60.0% vs. 13.8%; *p* < 0.001), longer recovery unit stay (2.80 ± 5.12 vs. 1.33 ± 0.96 days; *p* = 0.062), and markedly longer total in-hospital stay (11.2 ± 14.9 vs. 4.9 ± 2.3 days; *p* = 0.001). The specific complications in the transfused patients were: four cases of surgical cervical bleeding requiring operative re-exploration, two cases of ischaemic stroke, and one peripheral nerve injury.

**Figure 1:**
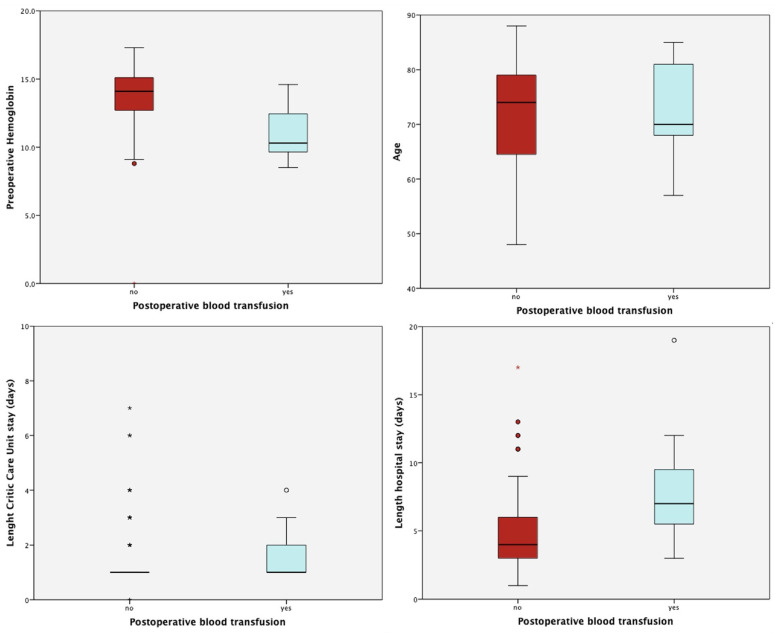
Predictors of postoperative blood transfusion on multivariate analysis (continuous variables): critical care unit stay (2.80 ± 5.12 vs. 1.33 ± 0.96 days; *p* = 0.062; RR 2.17, 95% CI 1.21–4.32), total in-hospital stay (11.2 ± 14.9 vs. 4.9 ± 2.3 days; *p* = 0.001; RR 4.22, 95% CI 2.15–7.28), age (71.2 ± 12.3 vs. 73.8 ± 10.8, *p* = 0.183; RR 1.79, 95% CI 1.09–3.25), and preoperative haemoglobin levels (14.2 ± 4.3 10.1 ± 3.2; *p* < 0.001; RR 3.42, 95% CI 1.98–5.67).

On univariate analysis, PAD was the strongest predictor of transfusion (60.0% vs. 22.8%; *p* = 0.002; RR 5.09, 95% CI 1.70–15.21). Lower preoperative Hb was significantly associated with transfusion need (10.99 ± 1.78 vs. 13.78 ± 2.04 g/dL; *p* < 0.001) (Table 3). Diagnostic workup by CT angiography was protective against transfusion (RR 0.17, 95% CI 0.05–0.57), whilst angiographic workup was associated with higher transfusion risk (RR 3.40, 95% CI 1.16–9.95). On multivariate analysis, independent predictors of postoperative transfusion were preoperative Hb (*p* < 0.001), PAD (*p* = 0.050), postoperative complications (*p* = 0.004), and length of hospital stay (*p* < 0.001) (Figure 1).

## 4. Discussion

This study demonstrates that postoperative blood transfusion is an independent predictor of 30-day mortality after CEA, conferring an approximately five-fold increase in risk. The association held on multivariate analysis independently of other complications, and the transfusion rate of 8.2% is considerably higher than rates reported in large registry series. Rubinstein et al. reported a transfusion rate below 1% in their series of elective CEA patients at a major US academic centre, attributing the low figure to strict intraoperative haemostatic technique and a highly selected patient cohort [5]. In contrast, the AlSheikh et al. single-centre retrospective study reported outcomes in a mixed CEA population with a similarly high anaesthetic risk burden, observing 30-day mortality and complication rates broadly comparable to our own findings [4]. The higher transfusion rate in our cohort most likely reflects the relatively liberal haemoglobin trigger applied (Hb ≤ 9 g/dL), the high proportion of ASA III patients (78.6%), and the absence of a structured patient blood management protocol during the study period.

Angeletti et al., in a risk stratification study of CEA patients, identified preoperative haemoglobin as a key determinant of one-year adverse events, reinforcing the prognostic importance of baseline haematological status in this population [13]. The 30-day mortality rate of 3.8% must be contextualised against the predominantly ASA III risk profile; the stroke rate of 6.0% is higher than registry benchmarks but is consistent with a cohort including a substantial proportion of patients with prior stroke (33.0%) and contralateral carotid occlusion (11.0%). Kline et al. reported a 30-day major morbidity and mortality rate of 4.1% in a propensity-matched CEA cohort with comparable comorbidity burden, with postoperative bleeding as the principal complication requiring re-intervention [17].

The independent association between transfusion and mortality is biologically plausible. Storage of allogeneic red cells beyond a range of 14–21 days results in the progressive loss of 2,3-diphosphoglycerate and adenosine triphosphate, impairing erythrocyte deformability and oxygen release at the tissue level [7]. Bioactive lipids, cytokines, and extracellular vesicles accumulating during storage promote systemic endothelial inflammation and microcirculatory dysfunction upon transfusion. In the cerebrovascular context, the resulting increase in blood viscosity may directly increase the risk of perioperative stroke and haemodynamic ischaemia. Rubinstein et al. demonstrated precisely this mechanism in CEA patients, reporting that the transfusion of one or two units of packed red cells was associated with a five-fold increase in stroke risk—a magnitude almost identical to the mortality risk amplification observed in our study [5]. Obi et al., in a large multi-institutional analysis of major vascular surgery patients, confirmed that perioperative transfusion was an independent predictor of 30-day mortality (odds ratio: 1.65), myocardial infarction, and pneumonia, and advocated for restrictive strategies except in patients with active haemorrhage or symptomatic anaemia [9]. The recent TOP randomised trial by Kougias et al., which enrolled 1428 veterans undergoing major vascular or general surgery, found no significant difference in a composite outcome of death, myocardial infarction, coronary revascularisation, acute kidney injury, and stroke at 90 days between liberal (Hb trigger < 10 g/dL) and restrictive (Hb trigger < 7 g/dL) strategies (9.1% vs. 10.1%; RR 0.90, 95% CI 0.65–1.24), suggesting that individualised decision-making based on clinical signs of anaemia-related hypoxia rather than a fixed haemoglobin threshold may represent the optimal approach in high-cardiovascular-risk patients [10].

The meta-analysis by Hovaguimian and Myles found that restrictive strategies in cardiac and vascular surgical patients may increase the risk of events reflecting inadequate oxygen supply (RR 1.09; 95% CI 0.97–1.22) and mortality (RR 1.39; 95% CI 0.95–2.04), contrasting with neutral or beneficial effects seen in general and critical care populations [11]. The TRICS III trial demonstrated non-inferiority of a restrictive strategy (trigger Hb < 7.5 g/dL) versus a liberal one (trigger Hb < 9.5 g/dL) in high-risk cardiac surgery, with fewer patients transfused in the restrictive arm and no increase in the composite of mortality, myocardial infarction, stroke, or renal failure [12]. Taken together, these data argue against a universally restrictive or universally liberal approach, and instead support an individualised strategy informed by clinical anaemia symptoms, haemodynamic stability, and organ-specific perfusion requirements—a position endorsed by Rao and Sherwood in their review of transfusion practice in cardiovascular patients [7].

The identification of PAD as the strongest independent preoperative predictor of transfusion (RR 5.09, 95% CI 1.70–15.21) has direct implications for pre-admission preparation. Patients with PAD represent a phenotype of diffuse systemic atherosclerosis characterised by greater baseline anaemia—attributable to chronic inflammation, nutritional deficiency, and renal impairment—impaired haematopoietic reserve, and higher operative complexity. Preoperative identification of this subgroup should prompt a structured assessment encompassing full blood count, iron studies, vitamin B12, and folate, followed by the targeted treatment of any correctable deficiency. Mandal et al. emphasised that perioperative anaemia management—including pre-admission iron supplementation and erythropoiesis-stimulating agents where indicated—significantly reduces allogeneic transfusion exposure and its associated morbidity across major surgical specialties [6]. The ICCAMS consensus recommends initiating preoperative anaemia correction four to six weeks before elective vascular surgery; combination therapy with intravenous iron, erythropoietin alpha, vitamin B12, and folate has been shown effective even for patients with limited pre-admission optimisation windows [15].

Low preoperative Hb was a further significant independent predictor of transfusion (*p* < 0.001). In our cohort, mean preoperative Hb in transfused patients was 10.99 ± 1.78 g/dL versus 13.78 ± 2.04 g/dL in those who did not require transfusion—a clinically significant difference of approximately 2.8 g/dL. Almonacid-Cardenas et al. similarly found that the optimisation of preoperative anaemia through iron therapy was associated with meaningful reductions in major postoperative complications and allogeneic transfusion rates in non-cardiac surgical patients, reinforcing the primacy of preoperative haemoglobin as the key modifiable determinant of transfusion need [14]. The protective association of CT angiography with transfusion risk (RR 0.17) most likely reflects a case-mix effect: patients evaluated with this modality represent a lower-risk phenotype, whilst those requiring digital subtraction angiography (RR 3.40) tend to have more severe or anatomically complex lesions. Accordingly, diagnostic modality should be interpreted as a surrogate for disease complexity rather than a direct transfusion determinant.

The need for postoperative transfusion is not unique to this study or exclusive to CEA, as several published studies on the use of CAS also demonstrate the need for postoperative transfusions. For instance, Qureshi et al. [18] analysed a large cohort of 20.645 patients treated with either CEA (*n* = 9.455) or CAS (*n* = 11.190), reporting postoperative transfusion rates of 4.05% and 3.65% in the CAS and CEA groups, respectively. In another study published in 2021 [19], including 17.429 patients evaluating the use of protamine after CAS, a propensity score-matched analysis of 2.300 patients yielded a postoperative transfusion rate of 1.13%.

### Limitations

Several limitations merit consideration. The retrospective single-centre design introduces selection and ascertainment bias, and the modest cohort size limits statistical power for subgroup analyses and precluded the application of propensity-score methods for confounding adjustment. No standardised transfusion protocol was in place during the study period; decisions were made at individual clinician discretion, likely contributing to practice heterogeneity and a higher-than-expected transfusion rate. Follow-up was restricted to the index admission, precluding an assessment of longer-term cerebrovascular and cardiovascular outcomes beyond 30 days. The haemoglobin trigger applied (Hb ≤ 9 g/dL) is more liberal than the thresholds range of 7–8 g/dL recommended by contemporary guidelines for haemodynamically stable patients, which may have inflated the observed transfusion rate and amplified its association with adverse outcomes. Neither the postoperative haemoglobin level nor the magnitude of haemoglobin drop was formally included in the multivariate models. Missing data were handled by complete-case analysis without formal imputation. Unmeasured confounders—including intraoperative blood loss volume, surgeon experience, and postoperative medication protocols—were unavailable. Future prospective, multicentre studies with predefined restrictive transfusion protocols and machine-learning-assisted outcome prediction—as proposed by Li et al. in a recent CEA cohort—are needed to validate and extend these findings [20].

## 5. Conclusions

Postoperative blood transfusion was independently associated with 30-day mortality after carotid endarterectomy in this observational cohort, with an approximately five-fold increase in risk, although this estimate should be interpreted with caution given the small number of mortality events. Peripheral arterial disease and low preoperative haemoglobin were the main preoperative determinants of transfusion requirement. These findings are exploratory and hypothesis-generating; prospective studies with larger sample sizes are needed to confirm these associations and establish causality. Nevertheless, the results highlight the potential clinical relevance of systematic preoperative anaemia assessment—including iron studies and targeted correction—particularly in patients with PAD, and the importance of developing evidence-based institutional transfusion protocols for patients undergoing carotid surgery. Prospective multicentre studies with standardised transfusion protocols and extended follow-up are needed to define optimal haemoglobin thresholds and determine whether reducing transfusion exposure translates into improved long-term outcomes after CEA.

## Figures and Tables

**Table 1:** Baseline characteristics and cardiovascular risk factors of the study population. COPD: chronic obstructive pulmonary disease; CEA: carotid endarterectomy; ASA: American Society of Anesthesiologists.

Variable	*n* = 182	Survivors (*n* = 175)	Non-Survivors (*n* = 7)	*p*
Male sex	159 (87.4%)	152 (86.9%)	7 (100.0%)	*0.305*
Mean age, years (range)	71.8 (48–88)	70.2 (48–86)	71.9 (54–88)	*0.156*
Hypertension	117 (64.3%)	111 (63.4%)	6 (85.7%)	*0.228*
Dyslipidaemia	99 (54.4%)	94 (54.3%)	5 (71.4%)	*0.371*
Diabetes mellitus	70 (38.5%)	66 (37.7%)	4 (57.1%)	*0.300*
Active smoker	46 (25.3%)	46 (26.3%)	0 (0.0%)	*0.285*
Ex-smoker	63 (34.6%)	61 (34.9%)	2 (28.6%)	*0.175*
Ischaemic heart disease	47 (25.8%)	46 (26.3%)	1 (14.3%)	*0.477*
Peripheral arterial disease	47 (25.8%)	44 (25.1%)	3 (42.9%)	*0.294*
COPD	19 (10.4%)	19 (10.9%)	0 (0.0%)	*0.357*
Chronic kidney disease	15 (8.2%)	14 (8.0%)	1 (14.3%)	*0.553*
Prior contralateral CEA	25 (13.7%)	25 (14.3%)	0 (0.0%)	*0.282*
ASA III/IV anaesthetic risk	148 (81.3%)	141 (80.5%)	7 (100.0%)	*0.124*
Dacron patch closure	159 (87.4%)	152 (86.9%)	7 (100.0%)	*0.305*
Severe ipsilateral stenosis (70–99%)	161 (88.5%)	160 (93.3%)	1 (85.7%)	*0.538*
Asymptomatic presentation	65 (35.7%)	63 (36.0%)	2 (28.6%)	*0.688*

**Table 2:** Postoperative variables comparing survivors and non-survivors. Hb: haemoglobin. Bold *p* values indicate statistical significance (*p* < 0.05).

Variable	Survivors (*n* = 175)	Non-Survivors (*n* = 7)	*p*
Postoperative Hb (g/dL)	11.87	10.79	*0.573*
Postoperative platelets (×10^3^/mm^3^)	197.49	185.57	*1.000*
Recovery unit stay (days)	1.4 ± 1.1	3.0 ± 1.4	***0.001***
Total hospital stay (days)	5.4 ± 1.6	6.1 ± 2.9	*0.997*
Blood transfusion	7.4%	28.6%	*0.046*
Any postoperative complication	14.3%	100.0%	***<0.001***

**Table 3:** Baseline characteristics and preoperative details of patients undergoing carotid endarterectomy with or without postoperative transfusion. COPD: chronic obstructive pulmonary disease; CEA: carotid endarterectomy; ASA: American Society of Anesthesiologists. Bold *p* values indicate statistical significance (*p* < 0.05).

Variable	Transfused (*n* = 15)	Non-Transfused (*n* = 167)	*p*
Male sex	86.67	87.43	*0.707*
Diabetes mellitus	60.00	36.53	*0.073*
Hypertension	80.00	62.87	*0.572*
Dyslipidaemia	46.67	55.69	*0.592*
Smoking habit	53.33	60.48	*0.539*
Ischemic heart disease	46.67	23.95	*0.054*
COPD	6.67	10.78	*0.618*
Chronic kidney disease	20.00	7.19	*0.084*
Haematological disease	6.25	2.99	*0.445*
Peripheral arterial disease	60.00	22.75	***0.002***
Prior contralateral CEA	13.33	13.77	*0.092*
**Clinical presentation**			
Asymptomatic	60.00	33.53	***0.040***
Amaurosis fugax	6.67	6.59	*0.990*
TIA	13.33	25.75	*0.286*
Stroke	20.00	33.53	*0.283*
Resolved stroke	26.67	47.90	*0.114*
Ipsilateral stenosis	93.33	88.02	*0.538*
**Contralateral stenosis**			
0%	40.00	40.34	*0.361*
<30%	20.00	19.16	*0.937*
30–50%	13.33	24.55	*0.327*
50–70%	20.00	8.38	*0.139*
>70%	6.67	7.19	*0.940*
Occlusion	0.00	11.98	*0.155*
Preoperative Hb (d/dL)	10.99 (±1.78)	13.78 (±2.04)	***0.0001***
Preoperative platelets (10^3^/mm^3^)	231.27 (±74.24)	220.35 (±69.53)	*0.534*
**ASA classification**			
ASA II	0.00	20.36	*0.053*
ASA III	100	76.65	***0.035***
ASA IV	0.00	2.99	*0.497*
**Patch type**			
None	0.00	2.40	*0.544*
Dacron	73.33	88.62	*0.088*
PTFE	20.00	9.49	*0.110*
Pericardium	6.67	0.60	***0.031***
Vein	0.00	0.60	*0.764*

## Data Availability

The data underlying this article will be shared upon reasonable request from the corresponding author.
